# Spherical Polyelectrolyte Brushes as Flocculants and Retention Aids in Wet-End Papermaking

**DOI:** 10.3390/molecules28247984

**Published:** 2023-12-07

**Authors:** Na Su

**Affiliations:** Department of Printing and Packaging Engineering, Shanghai Publishing and Printing College, Shanghai 200093, China; suna@whu.edu.cn

**Keywords:** spherical polyelectrolyte brushes, flocculation, retention aids, wet-end papermaking

## Abstract

As the criteria of energy conservation, emission reduction, and environmental protection become more important, and with the development of wet-end papermaking, developing excellent retention aids is of great significance. Spherical polyelectrolyte brushes (SPBs) bearing polyelectrolyte chains grafted densely to the surface of core particle have the potential to be novel retention aids in wet-end papermaking not only because of their spherical structure, but also due to controllable grafting density and molecular weight. Such characteristics are crucial in order to design multi-functional retention aids in sophisticated papermaking systems. This review presents some important recent advances with respect to retention aids, including single-component system and dual-component systems. Then, basic theory in papermaking is also briefly reviewed. Based on these advances, it emphatically describes spherical polyelectrolyte brushes, focused on their preparation methods, characterization, conformation, and applications in papermaking. This work is expected to contribute to improve a comprehensive understanding on the composition, properties, and function mechanisms of retention aids, which helps in the further investigation on the design of novel retention aids with excellent performance.

## 1. Introduction

The wet-end of papermaking is a very complex system, where paper stock is a suspension based on fibers with water as the medium. The components of paper stock are relatively complex [1], mainly including the following components: (a) Fibers. Based on the source of raw materials, these can be divided into wood pulp, reed pulp, straw pulp, etc. The chemical components mainly include cellulose, hemicellulose, and lignin. (b) Granular fillers. These consist of inorganic fillers such as ground calcium carbonate (GCC), precipitated calcium carbonate (PCC), kaolinite, talc powder, titanium dioxide, etc., and organic polymer fillers such as polyethylene fillers, fiber fillers, etc. The filler particle size is generally less than 10 μm [2]. (c) Various additives. These can be divided into two categories [3]: process additives and functional additives. The former includes retention aids, filter aids, defoamers, fungicides, etc., with a focus on improving economic benefits [4,5]. The latter is mainly designed to improve the end-use properties of papers, involving sizing agents [6], dry strength agents [7], wet strength agents, whitening agents, etc. (d) Soluble inorganic salts and other impurities. 

**Figure 1:**
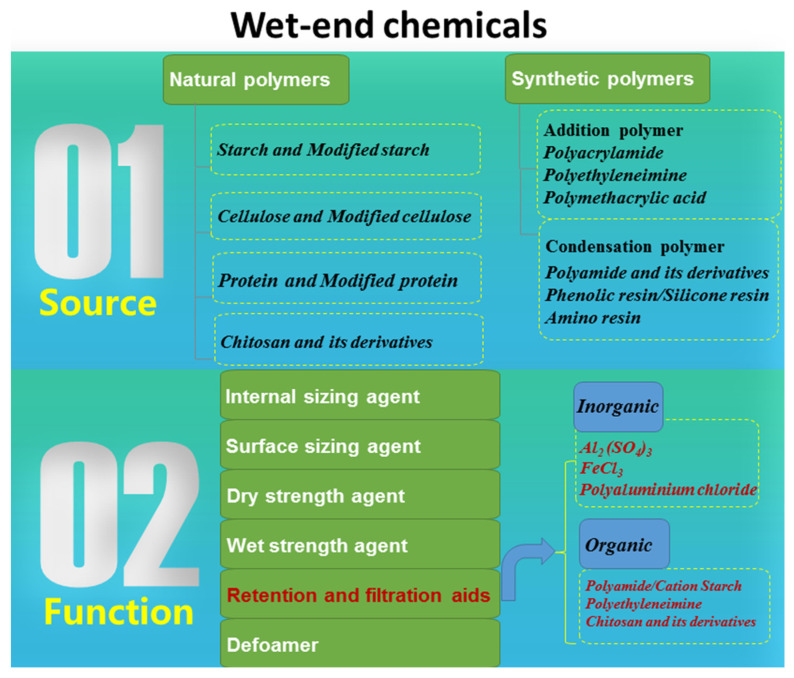
Classification of wet-end chemicals.

The development of the papermaking industry has put forward higher requirements for the application of wet-end chemicals in papermaking. Two application purposes of wet-end chemicals are mainly involved. One is to gain access to various properties of paper. The other is to increase production efficiency and improve the operation of the paper machine. Accordingly, wet-end additives are mainly divided into two categories: functional additives and process additives. The classification of wet-end chemicals in papermaking is shown in Figure 1. 

Over the past several years, intense research on new types of retention aids has resulted in an enormous number of fundamental [8,9,10,11] and applied studies [12,13,14,15] due to their significant effect on paper stock quality, and the operation efficiency of paper machines. The retention efficiencies of retention aids are of great significance, especially under high shear condition. The appropriate use of retention aids is an effective way to reduce environmental pollution and save resources via the retention of fines and fillers. 

Spherical polyelectrolyte brushes (SPBs) refer to the system that long polyelectrolyte chains are grafted to colloidal spheres [16]. Recently, it has attracted much attention due to its excellent retention performance in papermaking originating from its core–shell structure [17]. It not only takes the advantage of the ionization surface and three-dimensional nanostructures, but also has the controllable charge density and molecular weight of grafting polymer chains [18,19,20]. Moreover, its spherical symmetry or quasi-symmetric structure has the ability of shear resistance. So the flocs flocculation would be easier to be induced by SPBs though the bridging effect, thus making the flocs stronger in turbulence. Consequently, considered as an effective retention aid, SPBs have a promising application prospect in wet-end papermaking.

To date, a series of reviews about retention aids in wet-end papermaking have focused on chitosan [21], cellulose [22], and polymer [23]. As for SPBs, since the first review reported by Ballauff, M., 2007 [24], many published reviews have mainly concentrated on the construction of nanocomposites [25], ionic effects [26] and their application in sustainable energy [27], and nanoreactors [28]. However, there is no review related to SPBs as a retention aid in wet-end papermaking, which is still needed. In this article, recent studies on the retention aids and basic theory of wet-end papermaking are reviewed firstly, including single-component systems and dual-component systems as well as their application on papermaking. Furthermore, the synthesis and application of SPBs in wet-end papermaking, and the interaction of SPBs with calcium carbonate and fibers, are described. Through this review, a new direction in retention aids in wet-end papermaking, where we see great potential for SPBs, is highlighted, emphasizing the structure–function relationship with respect to retention performance. Finally, the existing challenges and development trends of high performance retention aids are proposed and prospected. 

## 2. Retention Systems

The retention of fine colloidal particles (e.g., fine fibers and filler particles) on the mesh in the slurry without additives is caused by mechanical interception. Therefore, the retention rate is mainly determined by the size of the interstitial space in fibers. In practical industrial production, a large amount of filler and fine fibers are lost in white water through the mesh due to high turbulence and high shear forces, resulting in a relatively low retention rate. Therefore, it is often necessary to add retention aids to improve the retention rate of fine colloidal particles in the slurry on the mesh. Retention aids can change the aggregation behavior of hydrophobic colloidal. With the continuous development of wet-end papermaking systems, retention aids are also constantly improved. According to the composition of retention aids, commonly used retention systems are single-component systems and dual-component systems. 

### 2.1. Single-Component Systems

Classical single-component systems are inorganic and organic, among which organic retention aids can be further divided into two categories: natural polymers and synthetic polymers.

Commonly used inorganic retention aids include Al_2_(SO_4_)_3_ [29,30], polyaluminium chloride (PAC) [31,32], and FeCl_3_ [33,34]. Due to the existence of a negative charge on the surface of paper pulp, the electroneutralization of multivalent metal ions results in flocculation [35,36]. Aluminum salts in water have complex structures and ion characteristics with changes in pH values and changing conditions, resulting in different adsorption properties on the surface of paper pulp [37]. Masato Kato et al. [38] investigated the retention performance of Al_2_(SO_4_)_3_ in bleached wood pulp. It was found that oxalic acid or Mg^2+^ reduced the content of aluminum, which was hardly affected by ionic strength. However, the shear resistance of the flocs in turbulence is limited, making it difficult for inorganic flocculants to meet the development needs of modern papermaking [39,40]. 

Natural polymers are widely used because of their characteristics of being cheap, renewable, and environmentally friendly. The commonly used natural polymers in papermaking are starch, chitosan, guar gum, and cellulose. Among these natural polymers, nature starch is by far the most used polymer due to its wide source and low price [41,42]. However, starch with low cationic degree (≤0.2) [43], which is available in many commercial areas, cannot provide sufficient attraction to stabilize adsorbed substances, resulting in its low retention [44]. So starch is usually modified physically or chemically to extend its usefulness. Svetlana Bratskaya et al. [45] reported a cationic starch with a cationic degree ranging from 0.25 to 1.54. They found that the cationic degree had a significant impact on retention performance, and the retention was significantly improved with increased cationic degree. Chitosan, as another type of natural polymer, has also been widely applied in papermaking [46,47,48,49]. Li et al. [50] studied the retention of modified chitosan in reed pulp/CaCO_3_, indicating that the maximum retention rate of CaCO_3_ could reach 80%. However, the low charge density and uncontrollable structure of natural polymers limit their application in papermaking. So efforts to overcome these drawbacks have led to a large amount of research attention focusing on the synthetic polymers.

**Figure 2:**
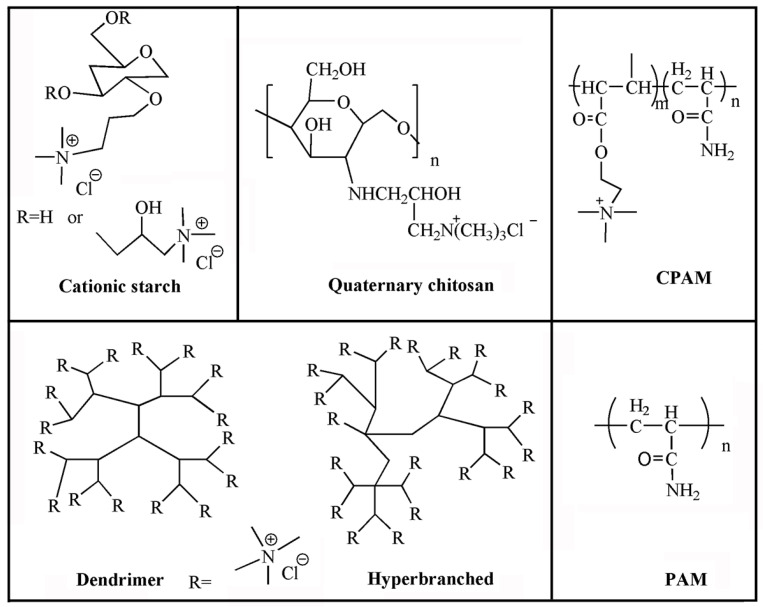
Structural diagrams of different polymer retention aids.

Currently, polyacrylamide (PAM) and polyethylene imine (PEI) are the most widely used synthetic polymers in papermaking, such as linear polyacrylamide [51], hyperbranched polyacrylamide [52], dendritic or star-shaped polyacrylamide [53], branched polyethylene imine [54], etc. The structure of polymer retention aids is shown in Figure 2. 

PEI is a multi-branched polymer with different weight and charge density. However, PEI unmodified by cationic activation is not suitable for alkaline papermaking [55,56,57]. It is because it loses charge when the pH is above 5.5. Carrasco et al. [58] studied the performance of cationic PEI in bleached wood pulp using zeta potential measurement and colloidal titration method. It was found that the presence of electrolytes had a negative effect on flocculation. Better retention effect of paper material was achieved when zeta potential fluctuated between −5 mV and 5 mV, but the shear resistance of PEI was not improved. 

Based on charge characteristics, polyacrylamide (PAM) is divided into four types: cationic polyacrylamide (CPAM), anionic polyacrylamide (APAM), amphoteric polyacrylamide (ACPAM), and nonionic polyacrylamide (NPAM). APAM and NPAM are mainly used in acidic papermaking process, so their application scope is restricted [59]. Due to the fact that paper pulps are generally negatively charged, CPAM has been applied in single-component retention systems [60,61,62,63]. The retention effect of CPAM is influenced by its charge density and molecular weight. ACPAM is widely used in papermaking owing to its anti-polyelectrolyte effect [64,65,66,67], which means its viscosity increases with the increase in salt ion concentration. Lu et al. [68] prepared ACPAM with different structures by inverse emulsion polymerization and found that favorable retention of fillers in the pulp was obtained when the anionic degree was 5%, the cationic degree was 20%, and the molecular weight was 200,000–300,000. 

Recently, hyperbranched polyacrylamide has attracted considerable interest because of its characteristics as a coagulant aid originating from its high cationic density and low molecular weight [69,70,71,72,73]. The formed flocs have the advantage of small volume, dense structure, and strong shear resistance. Further studies highlighted that branched polymers have higher retention and shear resistance than those of linear polymers [74,75,76]. Angeles Blanco et al. [77] studied the flocculation process of hyperbranched PAM on paper pulps using focused beam reflectance measurement (FBRM). It was noted that the charge density had a significant impact on the properties of the flocs. Low charge density had a negative impact on the growth of polyelectrolyte chain, thereby affecting the flocculation process, while high charge density may reduce the proportion of bridging mechanism and increase the proportion of patching mechanism. Polyacrylamide with a high molecular weight and hyperbranched structure has good application prospects in papermaking.

Additionally, dendrimer or star polymers have gradually become research hotspots as retention aids in papermaking due to their highly branched three-dimensional morphology [78,79,80,81]. Compared with linear polymers, they usually exhibit high solubility, low solution viscosity, small hydrodynamic radius, high shear resistance, small molecular volumes, and high-density functional groups [82]. Shan et al. [83] synthesized star-shaped CPAM by reversible addition–fragmentation chain transfer polymerization, which is a method of acrylamide and methacryloyloxyethyltrimethylammonium chloride copolymerizion. The retention rate of fillers and fine fibers was improved through bridging mechanism. 

For cationic microparticles, whether organic or inorganic particles, few conformational changes in cellulose flocculation occur because of their rigid structure [84,85,86]. Zegui Yan et al. [87] explored the retention performance of cationic SiO_2_ particles and cationic organic polymer particles in unwashed bleached kraft softwood pulp/PCC and found a significant improvement in retention rate. Xiaohong Peng et al. [88] prepared copolymer particles of acrylamide, acyloyloxyethyltrimethyl ammonium chloride (DAC), and polyethylene oxide (PEO) through inverse emulsion polymerization, and studied the retention effect of DAC and PEO on the fiber. Results indicated that the increase in the ratio of DAC and the chain length of PEO is beneficial to bridging, thereby improving the retention rate.

In summary, there are several theoretical models, namely “Electric neutralization”, “Patching”, and “Bridging”, that describe retention mechanism of retention aids. The neutralization effect is mainly caused by simple electrolytes and low-molecular-weight polyelectrolytes, such as Al_2_(SO_4_)_3_, FeCl_3_, and PEI. The patching effect is mainly caused by cationic polyelectrolytes with high charge density and medium molecular weight (100,000 to 1 million) (e.g., PEI and PAM). For high-molecular-weight polyelectrolytes, bridging effects have higher possibility than that of low-molecular-weight ones. 

### 2.2. Dual-Component Systems

With the continuous development of papermaking, existing single-component systems have been increasingly unable to meet the needs of retention aids under high shear conditions. In order to obtain flocs with higher shear resistance, great attention has been paid to develop dual-component systems. The process of dual-retention systems generally refer to the addition of cationic additives first, followed by the addition of anionic additives. 

In general, dual-component systems mainly involve two different systems: “dual polymeric retention system” and “microparticle retention-aid system”. The former is composed of fixatives with a low molecular weight and high charge density, and flocculants with a high molecular weight and low charge density. This system can achieve a relatively good retention effect, but the paper forming performance is limited by forming large and loose flocs. The latter system, the so-called “microparticle retention-aid system”, has developed into one of the most successfully commercially available products. Compared to traditional retention systems, it not only has excellent retention effects on wet-end chemical additives, fine fibers, and fillers, but also reduces the concentration of white water and increases the paper machine speed, thus generating environmental and economic benefits [89,90]. Interestingly, even if the adding order of binary components is reversed, there is almost no difference in its flocculation index. Classical microparticle retention aids are anionic particles and cationic particles.

Typical anionic particles are composed of the Compzil system (starch-colloidal silicon) [91] and the Hydrocol system (CPAM-bentonite) [92]. B. Alince et al. [93] studied the deposition behavior of calcium carbonate (PCC) on paper fibers in CPAM-bentonite systems. By changing the dosage of CPAM, it was found that CPAM provided an anchor point for bentonite to function on the fibers and PCC. Bentonite played a bridging role between the fibers and PCC. Excessive dosage of CPAM would weaken the bridging effect of bentonite. The interaction force between fibers and PCC obtained through the CPAM-Bentonite system was stronger than that of the CPAM system. Norlito Cezar et al. [94] reported cationic particles by modifying the surface of SiO_2_ with 2, 3-epoxypropyl H-methylammonium chloride. Results showed that it significantly improved the flocculation efficiency of clay. The optimal dosage ratio of cationic (SiO_2_) to anionic (APAM) was in the range of 2:1 and 5:1. Moreover, the rigidity of SiO_2_ improved the efficiency of bridging. Cationic magnesium aluminum hydroxide/APAM was used by Wang Songlin et al. to study its retention performance on talc powder/reed pulp. The retention effect improved with the decrease in colloidal particle size of magnesium aluminum hydroxide particles. The retention rate was highest at the conditions of the amount of magnesium aluminum hydroxide 0.6% and the amount of APAM 0.06%. 

In addition, organic polymer particles not only have the three-dimensional nanostructure of the ionized surface of inorganic particles, but also possess the controllable charge density and flexible chains of organic polymers, which is expected to be a novel retention aid [95,96,97,98]. Taking cationic organic polymer particles as an example, Xiao H et al. [99] prepared cationic organic particles by emulsifier-free emulsifier polymerization and studied their flocculation on fine clay. It was found that the dosage ratio of cationic particles and anions was 8:1 at the optimal flocculation point, and the dosage of anions accounted for 0.06 wt% of clay. These binary components played a synergistic role in the flocculation process. Ono Hiroshi et al. [100] synthesized cationic polymer particles (CPMP) with different charge densities and sizes by emulsion and micro emulsion methods, and studied the effect of CPMP on positively charged and negatively charged calcium carbonate. It was found that CPMP had no obvious effect on positively charged calcium carbonate. For negatively charged carbonate, however, the opposite is true. The addition of APAM could improve the flocculation effect. 

## 3. Evaluation Methods of Retention Aids

For single-component system and dual-component system, there are a variety of ways to measure the retention efficiency, such as fluorescence microscopy, field emission electron microscopy (FE-SEM), transmission (TEM), focused beam reflectance measuring instrument (FBRM), flocculation turbidity, zeta potential, and dynamic retention. The Dynamic Drainage Jar (DDJ) is a widely used reliable instrument for testing the retention efficiency of single or multiple retention aids. The first-pass retention (*FPR*) of pulp suspension and PCC can be calculated based on Equation (1):

(1) $$FPR=\frac{C_{i}-C_{0}}{C_{i}}\times 100\%$$

where *C_i_* and *C*_0_ are the concentrations of colloidal particles in slurry and in filtrate, respectively.

## 4. Basic Theory in Wet-End Papermaking

Wet-end chemistry of papermaking mainly studies the interaction among various components of paper stock during retention, filtration, forming, and white water circulation, as well as their effects on the operational performance of paper machines and product quality. Interface chemistry is an important theory in wet-end chemistry of papermaking. Due to the complex composition of colloids in papermaking, although most fiber sizes are beyond the scope of colloids, the gaps in fibers belong to the colloid size. Therefore, papermaking process can be studied by the theories related to colloid and interface chemistry. 

### 4.1. Main Forces

The wet-end papermaking is a complex and diverse polydisperse system, in which there are various forces involved [101]. In terms of macroscopic components, it can be mainly summarized into following seven types: (l) adsorption of various additives on fibers, fine fibers, and fillers; (2) aggregation of fibers, fillers, and fine fibers; (3) aggregation among various additives; (4) interaction with water between fibers, fine fibers, and additives; (5) neutralization of surface charges of suspended and soluble anionic substances; (6) formation and development of micelles that comprise surfactant molecules; (7) establishment of dynamic equilibrium between soluble inorganic salts and insoluble electrolytes.

From the perspective of micro-force, the forces among main components mentioned above are manifested as van der Waals forces, hydrogen bonds, ionic bonds, and covalent bonds. As for drying paper, the strength of the forces among fibers varies: the hydrogen bond is 8.81 KJ/mol, the ionic bond is 41.8–54.34 KJ/mol, the covalent bond is 292.6–351.12 KJ/mol [102], and the van der Waals strength is weaker than the others.

### 4.2. Machinism of Interface Interaction

#### 4.2.1. The Conformation of Polyelectrolytes

**Figure 3:**
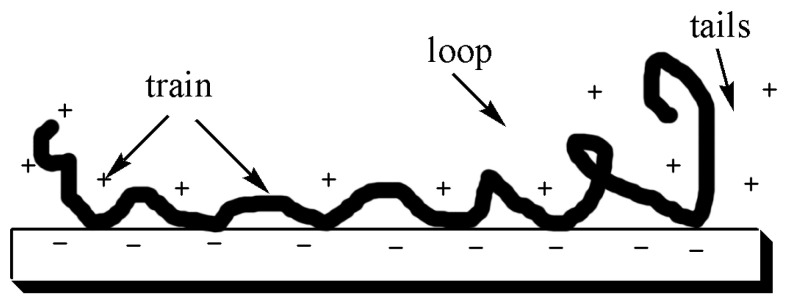
Conformation of adsorbed polyelectrolytes on solid surfaces.

The conformation of linear polyelectrolytes in solution changes continuously. Numerous studies have shown that polyelectrolytes mainly exist in the configuration of circular adsorption or flat [103,104,105,106]. For high-level potential energy surface‚ polyelectrolytes have a small molecular weight, high charge density, and strong electrostatic interactions. So polyelectrolytes are prone to exist on solid surfaces in a flat configuration. Figure 3 displays the adsorbed polyelectrolytes, which was divided into three segments by Böhmer et al. [107]: train, loop, and tails. The length of each segment depends on its charge density, molecular weight, negative charge on the solid surface, etc. For example, the stronger the electrostatic effect, the smaller the molecule‚ thus resulting in more trains‚ and fewer loops and tails. Theoretically, it is predicted that when polyelectrolyte chains driven by strong electrostatic forces are absorbed on the solid surface, they are composed of 80% trains‚ 20% loops‚ and rare tails.

#### 4.2.2. Adsorption between Polyelectrolytes and Fibers

**Figure 4:**
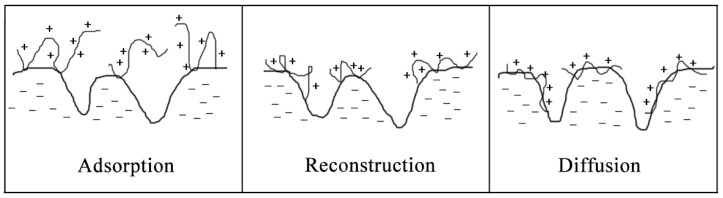
Schematic diagrams of adsorption, reconstruction, and diffusion of polyelectrolytes.

Generally speaking‚ the adsorption process between cationic polyelectrolytes and fibers can be divided into three stages [108]: adsorption, reconstruction, and diffusion, as shown in Figure 4. For cationic polyelectrolytes with different structures, the stages will vary.

For polyelectrolytes with low molecular weight and high charge density‚ they rapidly adsorb on the fiber surface in a flat configuration by ion-exchange mechanism. And it almost immediately diffuses into the fiber without any molecular reconstruction process. Cationic polyelectrolytes with medium molecular weight display a flat configuration during initial adsorption‚ resulting in low positive charge density on the surface of pulps. Subsequently, only minor reconstruction on the surface of pulps happens. Therefore, the charge decay at the second stage is also smaller than that of polyelectrolytes with high molecular weight. After adsorption of cationic polyelectrolytes with highly branched structures and high molecular weight on pulp fibers‚ the surface charge of fibers rarely decays. This is because it is difficult for the reconstruction and diffusion process of polyelectrolytes on the surface of pulp fibers to occur.

#### 4.2.3. Retention Mechanism

(a) Single-component systems

According to the theory of colloidal aggregation‚ the retention mechanism can be divided into three modes: coagulation, flocculation, and agglomeration. 

Coagulation implies that for negatively charged colloidal particles such as fine fibers, talc powder, calcium carbonate, titanium dioxide et al., the double layer becomes thinner and zeta potential falls under the action of electrolytes. Therefore, the electrostatic repulsion forces which commonly exist between colloid particles decrease‚ resulting in the instability of colloid particles. The retention is produced by the formation of small aggregates (also known as “soft aggregates”).

Colloidal particles are bonded together by polymers with a high molecular weight (greater than 100,000) due to the instability phenomenon [109]. The coagulant adsorbs onto the surface of colloidal particles in a random coil conformation through electrostatic or non-electrostatic forces. These adsorbed polymers form so-called “hard aggregates” by reacting with another colloidal particle. We call this process flocculation.

**Figure 5:**
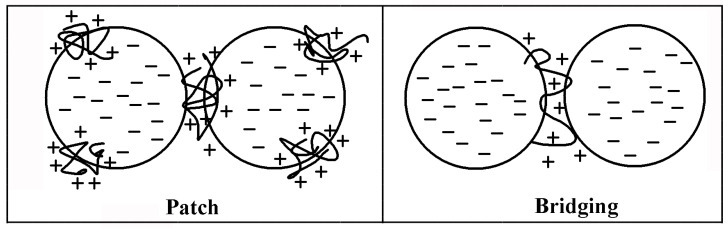
Mechanism diagrams of “Patch” and “Bridging”.

Agglomeration refers to the instability phenomenon of colloidal suspensions caused by the action of polymers with low molecular weight (less than 100,000) and high charge density. Due to the high positive charge density of retention aids‚ they adsorbed on the surface of negatively charged particles through ion bonds‚ forming so-called ‘patch’ [110]. These positive charge patches will agglomerate with the negative charge of another particle by “bridging” (see Figure 5).

(b) Dual-polymeric retention system

There are some defects in single-component systems. The formed flocs are sensitive to hydrodynamic forces, leading to flocs fragment exposed to turbulent shear forces. While the shear force slows down or disappears, these dispersed flocs will not be restored, making its retention ability greatly reduced. A novel retention system has emerged, which is so that that the synergistic effect is generally achieved through dual components [111].

Research has found that hard and tough flocs can be generated by combining two types of polyelectrolytes with different charges and controlling adding order. If a cationic polyelectrolyte is added before adding an anionic polyelectrolyte‚ the retention effect is far better than that of single-component systems [112]. The acting mechanism may be explained as follows: 

After adding cationic polyelectrolytes with low molecular weight and high charge density‚ polyelectrolytes are adsorbed by negatively charged particles in a flat configuration‚ causing cationic patches. These adsorbed polyelectrolytes only have very few loop and tails extending into the aqueous solution‚ but providing anchoring points for anionic polyelectrolytes. Anionic polyelectrolytes with high molecular weight and low charge density are then bonded onto cationic patches. However, many segments of anionic polyelectrolytes will extend into aqueous solutions due to the repulsive force of negative charges on the particle surface. They bind with cationic patches‚ just like a bridge‚ agglomerating small particles into large flocs.

(c) Microparticle retention-aid system

As previously mentioned, the dual-microparticle retention-aid system is currently recognized as the most advanced and effective retention system. It can be used in high-speed spray forming machines and fully enclosed circulation systems. 

The Compzil system was developed and applied in papermaking by a Swedish company. It is a control system composed of cationic starch and SiO_2_ nanoparticles (3–5 nm) with high specific surface area and high negative charge density. The retention mechanism of filler is generally believed to be that fine fibers and filler particles are aggregated by cationic starch, thus generating flocculent. When SiO_2_ nanoparticles are added, this flocculent may be torn apart into small flocs. For low-charge-density polyelectrolytes, the flocculation mechanism seems to be mainly the bridging effect [113]. As for polyelectrolytes with a high charge density, electrostatic attraction plays a major role. Obviously, due to the migration of SiO_2_ nanoparticles into the adsorbed cationic starch, the pulp and silicon particles form a network floc that has a tendency to reaggregate after shearing. Therefore, the uniformity of paper will not deteriorate even under high retention rate. In addition, the structure of finished paper will become loose, resulting in increased porosity and improved water filtration performance. 

The Hydrocol system is a typical duplex control system which was developed in the United Kingdom. The working principle may be that relatively coarse flocs are formed when cationic electrolytes with a high molecular weight are added into the pulp. However, these flocs will be cut into small flocs when it is subjected to the action of a mixing pump and a screening machine. These tiny flocs, under the action of Heidelberg pigments, will reaggregate into a uniform network flocculent. So filler particles or fine fibers are retained in the paper pulp to achieve the purpose of retention.

## 5. Spherical Polyelectrolyte Brushes 

**Figure 6:**
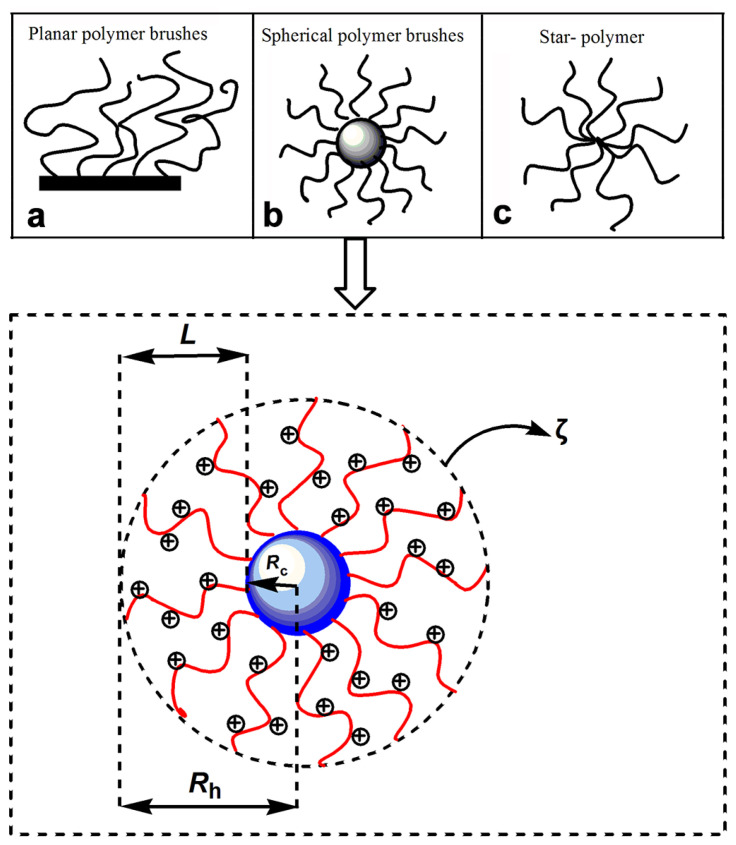
The structure diagram of polyelectrolyte brushes: (**a**) Planar polymer brushes, (**b**) Spherical polymer brushes, and (**c**) Star-polymer.

Polymer brushes [114] with unique properties such as a high charge density, high symmetry or quasi-symmetric structure, and low molecular weight provide great potential and limitless possibilities for its applications in biomedical applications [115,116,117], nanoreactors [118,119,120], durable protective clothing [121,122], etc. The brush conformation is influenced by the curvature of grafted surface. Figure 6 displays the structure of polymer brushes on different grafting surface. When the radius of colloidal particles is much greater than the thickness of the grafted brush layer, planar brushes form (see Figure 6a). The colloidal particle size is much less than the thickness of grafted brush layer, resulting in the formation of a star-polymer (see Figure 6c). If the polyelectrolyte chains affix to the sphere surface, then, e.g., spherical polyelectrolyte brushes (SPBs) result [25]. As shown in Figure 6b, the core radius R_c_ denotes initiator-immobilized spheres surface, from which polyelectrolyte chains are grafted. L refers to the thickness of the brush layer, R_h_ is the hydrodynamic radius, and ζ is the zeta potential. SPBs not only possess many advantages originating from their special molecular structure, but also has a simple synthesis process [123,124] and easy-to-control molecular weight [125,126]. Therefore, SPBs have recently attracted worldwide research interest [127,128,129,130,131,132]. 

### 5.1. Synthesis Methods

#### 5.1.1. Physisorption

**Figure 7:**
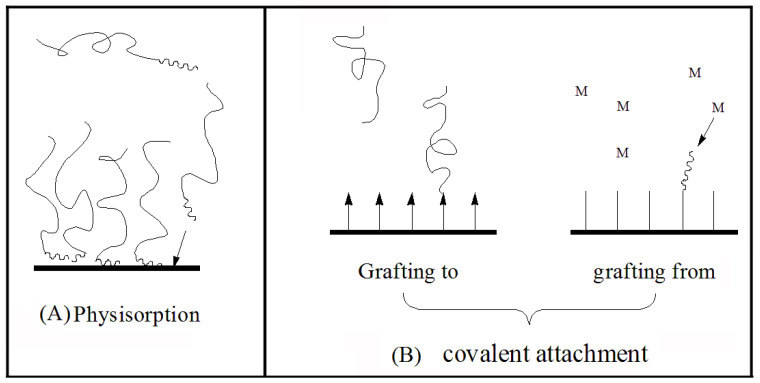
Schematic representation of physisorption (**A**) and covalent attachment (**B**).

Physisorption is a reversible process, which is the self-assembly process of macromolecules with surface activity or polymers with end group functional groups [133], as shown in Figure 7A. Both graft copolymers and block copolymers can be formed by physisorption. The essence of the process is based on selective solvation. The insoluble segments settle down and affix on the matrix, while the dissolved segments stretch, forming a polymer brush. Additionally, polymer brushes can also be formed when selective adsorption of graft copolymers occurs on the substrate surface.

Taking toluene as a selective solvent, Parsonage E et al. [134] studied the polymer brush formed by the adsorption of a PS-b-PVP copolymer with different molecular weight on the substrate. PVP was affixed to the substrate surface and PS formed a polymer brush. Motschman H et al. [135] discussed the adsorption kinetics and adsorption isotherms of PS-b-PEO copolymer on the surface of silica gel in toluene solvent. The results indicated that apparent two-rate processes were showed in adsorption kinetics in a certain time range. At first, weak interactions lied in the grafting chains due to the effect of surface diffusion. Subsequently, densely polymer brushes formed by osmosis behavior among grafting chains.

In the process of physisorption, desorbing of the adsorbed macromolecules happens easily due to the weak hydrogen bond or van der Waals force.

#### 5.1.2. Covalent Attachment

Covalent attachment is an irreversible process. That is, the polymer chains are connected by chemical bonds on substrate surface. This process can be realized by two technologies, namely “grafting to” and “grafting from”, as shown in Figure 7B.

“Grafting to” means that under appropriate reaction conditions, the synthesized polymers with functional end-groups will react with modified substrate surface, and then polymers covalently bonded to substrate surface to form a polymer brush [136,137]. Auroy P et al. [138] synthesized a PSS brush on the substrate surface of silica gel by a two-step method. Firstly, PS chain with trichlorosilane end-groups was grafted on the substrate surface by using the “grafting to” technology, and then the PSS brush was obtained by in situ sulfonation reaction on substrate surface. The shortcoming of the “grafting to” technique is that only a few kinds of polymers have been grafted to the substrate surface. Once the polymer chains on substrate surface have reached a certain amount, the remaining chains could no longer be grafted due to steric hindrance. In particular, as for the grafted polyelectrolyte chains, electrostatic repulsion should be considered. Therefore, this method has a negative influence on the grafting density of polymer brushes.

The “grafting from” technique is based on the surface being modified by polymerization initiators under light or heat [139,140,141,142]. The polymer brush prepared by this method is firmly bonded because of bearing initiator functionalities on substrate surface, thus leading to a dense layer of brush. Both conventional free radical polymerization and active free radical polymerization are employed to prepare polymer brush with high grafting density. 

Conventional free radical polymerization, by which the linear polymers are formed, is suitable for most vinyl monomers. So this method is the most widely used in the polymer industry. H and J [143] reported a poly (4-vinylpyridine) polymer brush created by free radical polymerization, and then the quaternization was commenced by adding CH_3_I to prepare a polyelectrolyte brush. When preparing brushes using acrylic acid as the monomer, it was found that increasing the reaction time and the solid content of the monomer were beneficial for increasing the length of brush. It is a simple and effective method for the synthesis of polymer brush, but low monomer utilization is a problem. It is mainly because that the two free radicals decomposed by an initiator are on the substrate surface and in the continuous phase, respectively, leading to a low monomer conversion rate (41.48%).

Active radical polymerization mainly includes two strategies: atom transfer radical polymerization (ATRP) [144,145,146,147] and reversible addition–fragmentation chain transfer (RAFT) [148,149,150,151]. Zhao B et al. [152] synthesized mixed polymer brushes of PS and PMMA using ATRP technology and nitroxide-mediated radical polymerization technology. The grafting density, molecular weight, and distribution of the mixed polymer brush are controllable. Baum M et al. [153] synthesized PS, PMMA, and PAA-co PMMA polymer brushes using RAFT technology by bonding nitrogen-containing initiators onto the surface of silicon. Due to the low concentration of surface initiators, additional initiators are required to increase polymerization rate.

### 5.2. Characterization and Conformation

So far, various experimental methods have been adopted to characterize the SPBs. For example, the morphology of SPBs can be characterized by cryogenic transmission electron microscopy (Cryo-TEM) [154,155,156,157], small-angle neutron and X-ray scattering [158,159,160,161]. The particle size can be measured by atomic force microscopy (AFM) [162,163,164] and dynamic light scattering (DLS) [165,166,167,168]. The molecular weight and distribution of polymer brushes can be characterized by gel permeation chromatography (GPC) [169,170,171], and then surface grafting density is calculated [172,173]. Curie E P K et al. [174] studied the polymer brush of hydrogenated PS-b-PEO diblock copolymer by the neutron reflection technique. It was found that chain density changed from step distribution to parabola distribution with the increase in grafting density. Guo x et al. [175] studied the hydrodynamic radius RH of micellar polyelectrolyte brush consisting of PS core (50~100 nm) and PAA brushes at different KCl concentration (10^−3^~3 m) by DLS. It was found that R_H_ was inversely proportional to the ionic strength, which was consistent with the Daoud–Cotton model. Prucker O et al. [176] studied a PS brush with different grafting densities grafted on silicon wafer by AFM. The results showed that the structure of the brush was dependent on the grafting density. When the grafting density was low, the surface was uneven because of the aggregation of PS chain. On the contrary, the collapse of the PS chain could form a uniform coating.

In order to explore the conformation of SPBs, many theoretical models have been explored [177]. According to the complexity of models, it can be divided into the following categories: scale theory [178,179,180], numerical self-consistent field theory (NSCFT) [181,182,183], analytical self-consistent field theory (ASCFT) [184,185], molecular dynamics (MD) [186], Brownian dynamics (BD) [187,188], and so on. Hariharan R et al. [189] studied an SPB system using the Daoud–Cotton model. Using the Monte Carlo model, Luo MB [190] studied the relationship between the conformational parameters of polymer brushes and the stretching volume of random and restricted chains. The results showed that the effects of the extended volume (EV) of two types of chains on the mean square end distance <R_2_> and the mean square radius of rotation <S_2_> are basically identical.

In addition, research on the fundamental nature of SPBs has been reported. Considering the special constitution of SPBs, the influence of external factors (ionic strength, pH) on the conformational parameters of polyelectrolyte brush can also be monitored by physical test methods. For example, Ballauff M [191] investigated the influence of pH value and ionic strength on brush thickness in a PS-PAA system. They found that the pH value of the system affected the dissociation degree of polyacrylic acid brush. In the acid condition, acrylic acid was difficult to dissociate. When NaOH was added to the system, OH^-^ was neutralized with H^+^, causing the presence of carboxylate anion on the acrylic acid chain. Due to the increase in the electrostatic repulsion force, the swelling of chains happens. Moreover, the effect of electrolyte concentration on the morphology of PAA brush on microspheres was mainly due to the electrostatic effect. The higher electrolyte concentration was, the more obvious the electrostatic shielding effect was, which led to the shrinkage of the polymer brush. Yu [192] found that the brush chain in the spherical PSS brush could fully swell in the water phase, and the brush collapsed after adding La^3+^, which may be caused by the decrease in osmotic pressure.

### 5.3. Application in Papermaking

**Figure 8:**
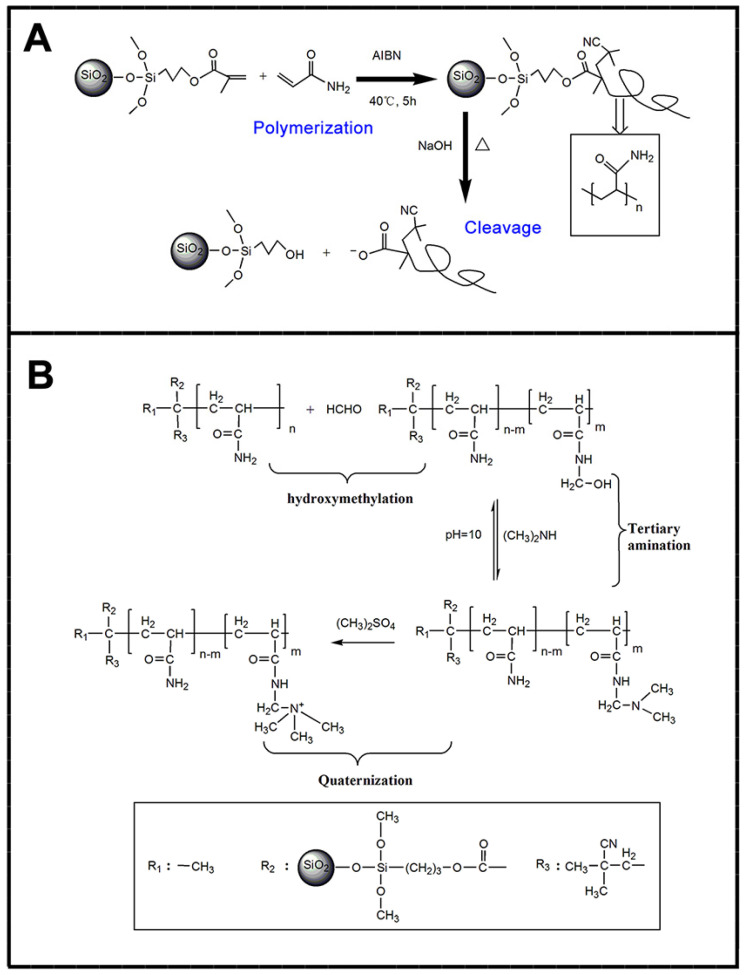
Schematic representations of synthesis process of SPBs (**A**) and cationization process of SPAM (**B**). Copied from De Gruyter [193].

As in our previous work (see Figure 8), cationic spherical polyacrylamide (CSPAM) brushes were synthesized by grafting manniched polyacrylamide (PAM) from the surface of γ-methacryloxypropyl trimethoxy-silane-modified SiO_2_ nanoparticles [193]. The retention effect of CSPAM revealed that the highest first-pass retention was 71.1% when the dosage of CSPAM was 3.5 mg·g^−1^.

**Figure 9:**
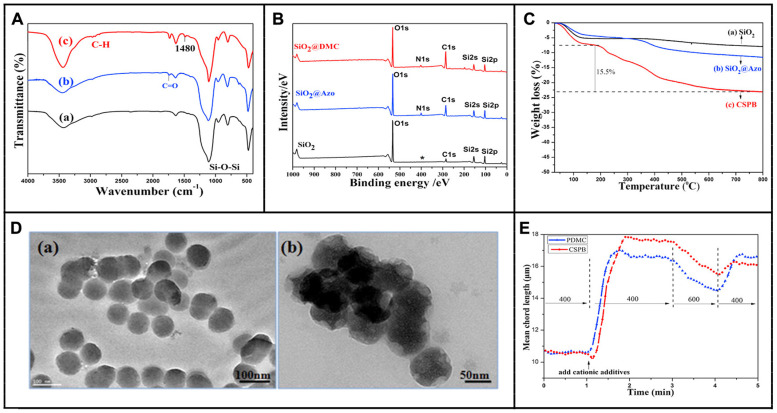
FTIR (**A**), XPS (**B**) (“*” denotes the signal position of nitrogen atoms in the spectrum of SiO_2_), and TGA (**C**) of CSPBs; TEM (**D**) of SiO_2_ (**a**) and CSPBs (**b**); mean chord length of pulp suspension induced by cationic additives (**E**). Reprinted with permission from [194]. Copyright: 2016, Elsevier.

Cationic spherical polyelectrolyte brushes (CSPBs), with poly (2-(acryloyloxy) ethyltrimethylammo-nium chloride) chains were grafted from the surfaces of colloidal silica particles by Zhang Xiongzhi et al. [194]. The CSPBs were characterized by various characterization methods involving Fourier-transform infrared spectrometry, thermo-gravimetric analysis, transmission electron microscopy, and X-ray photoelectron spectroscopy, as displayed in Figure 9. And then, the CSPBs were investigated as flocculation and retention-aids for bleached eucalyptus kraft pulp and kaolin/pulp particles. The results suggested that the flocculation and retention-aid properties were improved.

**Figure 10:**
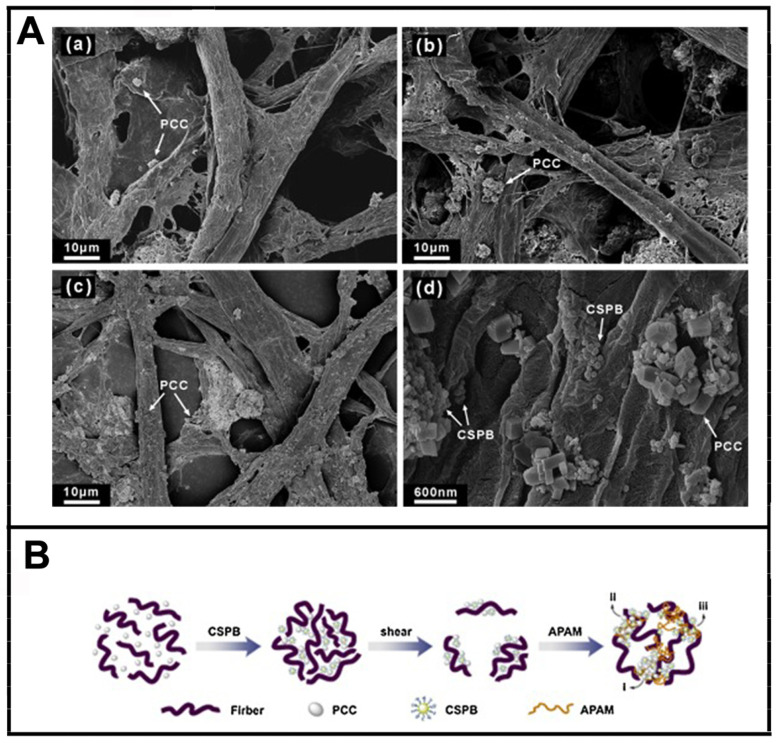
FSEM images of PCC flocs adhering to the surface of fibers (**A**): (**a**) Without any additives, (**b**) with cationic starch/APAM (cationic starch = 35 mg/g, APAM = 0.03 wt%), and (**c**,**d**) with CSPB-3/APAM (CSPB-3 = 18 mg/g, APAM = 0.03 wt%); Flocculation mechanism of the CSPB/APAM dual-component system (**B**). Reprinted with permission from [195]. Copyright: 2015, Elsevier.

Huang Yu et al. [195] reported a dual-component system consisting of CSPBs and anionic polyacrylamide (APAM) to improve the retention and drainage properties of bleached eucalyptus kraft pulp and precipitated calcium carbonate (PCC). CSPBs comprised a silica core and a shell of copolymer of acrylamide (AM) and [2-(methacryloyloxy) ethyl] trimethylammonium chloride (METAC). The CSPB/APAM system showed better retention efficiency than the cationic starch/APAM system under different turbulent conditions. As illustrated in Figure 10, the CSPB/APAM dual-component system interacted with fibers and PCC through a patching and bridging mechanism.

Y. Mei et al. [196] presented a comprehensive study of the interaction of SPBs with cationic modified polyacrylamide (CPAM), calcium carbonate (CaCO_3_) particles, and cellulose fibers. In their work, it also elaborated this system as a model for retention aids in the papermaking process. The results suggested that the dual flocculation system using anionic SPBs worked so efficiently compared to bentonite, which can be attributed to the high cation exchange capacity (CEC) of SPBs. Anionic SPBs acted as a particle bridge between fibers and CaCO_3_.

## 6. Conclusions and Outlook

As follows from the above overview, numerous retention aids have been utilized in wet-end papermaking. It can also be noticed that current studies are mainly focused on a basic description of single-component systems and dual-component systems, while much less attention is paid to the correlation of structure parameters involving charge density, molecular weight, and the retention performances of retention aids. Therefore, the prospect of developing novel retention aids with highly branched structures and controllable charge density and molecular weight is an exciting direction that has yet to be realized. Previous studies have found that spherical polyelectrolyte brushes can adapt to the current demand for papermaking.

Looking forward to the future, however, a wide scope of challenges will continue to create excellent performance retention aids due to the complexity of wet-end papermaking. Firstly, there are limited publications focusing on SPBs as retention aids in papermaking. In addition to CS and SiO_2_, other types of nanoparticles as templates should be further studied. More importantly, further development of SPBs with different grafting densities, molecular weights, and charge densities will be a key to realize the applications in wet-end papermaking. Secondly, current analysis involving the interaction force between CSPBs and fibers has been restricted to macroscopic analysis. That is to say, the specific interaction force, whether van der Waals or electrostatic forces, has not been clearly understood. Finally, the fractal dimension of flocs in wet-end papermaking and the retention mechanism of SPBs can also be further studied.

Interestingly, further research will continue to focus on multi-component additives.

## Data Availability

Data are contained within the article.

## Abbreviations

**A** **bbreviations**	**Name**
GCC	Ground calcium carbonate
PCC	Precipitated calcium carbonate
SPBs	Spherical polyelectrolyte brushes
PAC	Polyaluminium chloride
PAM	Polyacrylamide
PEI	Polyethylene
CPAM	Cationic polyacrylamide
APAM	Anionic polyacrylamide
ACPAM	Amphoteric polyacrylamide
NPAM	Nonionic polyacrylamide
FBRM	Focused beam reflectance measurement
DAC	Acyloyloxyethyltrimethyl ammonium chloride
PEO	Polyethylene oxide
DDJ	Dynamic Drainage Jar
CPMP	Cationic Polymer Microparticle
ATRP	Atom transfer radical polymerization
RAFT	Reversible addition–fragmentation chain transfer
PS	Polystyrene
PMMA	Polymethyl methacrylate
PAA	Polyacrylic acid
Cryo-TEM	Cryogenic transmission electron microscopy
DLS	Dynamic light scattering
AFM	Atomic force microscopy
GPC	Gel permeation chromatography
NSCFT	Numerical self-consistent field theory
ASCFT	Analytical self-consistent field theory
MD	Molecular dynamics
BD	Brownian dynamics
EV	Extended volume
CSPAM	Cationic spherical polyacrylamide
CSPBs	Cationic spherical polyelectrolyte brushes
AM	Acrylamide
METAC	2-(methacryloyloxy) ethyl trimethylammonium chloride
CEC	Cationic exchange capacity
